# Testing Mediation Effects in High-Dimensional Epigenetic Studies

**DOI:** 10.3389/fgene.2019.01195

**Published:** 2019-11-22

**Authors:** Yuzhao Gao, Haitao Yang, Ruiling Fang, Yanbo Zhang, Ellen L. Goode, Yuehua Cui

**Affiliations:** ^1^Division of Health Statistics, School of Public Health, Shanxi Medical University, Taiyuan, China; ^2^Division of Health Statistics, School of Public Health, Hebei Medical University, Shijiazhuang, China; ^3^Department of Health Sciences Research, College of Medicine, Mayo Clinic, Rochester, MN, United States; ^4^Department of Statistics and Probability, Michigan State University, East Lansing, MI, United States

**Keywords:** de-sparsify, DNA methylation, high-dimensional testing, high-dimensional mediation, mediation analysis

## Abstract

Mediation analysis has been a powerful tool to identify factors mediating the association between exposure variables and outcomes. It has been applied to various genomic applications with the hope to gain novel insights into the underlying mechanism of various diseases. Given the high-dimensional nature of epigenetic data, recent effort on epigenetic mediation analysis is to first reduce the data dimension by applying high-dimensional variable selection techniques, then conducting testing in a low dimensional setup. In this paper, we propose to assess the mediation effect by adopting a high-dimensional testing procedure which can produce unbiased estimates of the regression coefficients and can properly handle correlations between variables. When the data dimension is ultra-high, we first reduce the data dimension from ultra-high to high by adopting a sure independence screening (SIS) method. We apply the method to two high-dimensional epigenetic studies: one is to assess how DNA methylations mediate the association between alcohol consumption and epithelial ovarian cancer (EOC) status; the other one is to assess how methylation signatures mediate the association between childhood maltreatment and post-traumatic stress disorder (PTSD) in adulthood. We compare the performance of the method with its counterpart *via* simulation studies. Our method can be applied to other high-dimensional mediation studies where high-dimensional mediation variables are collected.

## Introduction 

Introduced by Baron and Kenny in 1986 (Baron and Kenny, 1986), mediation analysis has been broadly applied in many scientific disciplines, such as sociology, psychology, behavioral science, economics, epidemiology, public health science, and genetics (e.g., E.Shrout and Bolger, 2002; Preacher and Hayes, 2008; Hafeman and Schwartz, 2009; Pfeffer and Devoe, 2009; Imai et al., 2010; Rocca et al., 2010; Pearl, 2012; Pierce et al., 2014). Through solving a chain of relations between an exposure variable and an outcome, it helps to understand how the effect of one variable is transmitted to another variable. Thus, mediation analysis offers researchers a unique statistical tool to reveal the underlying mechanism or process of various scientific questions, especially when designing an intervention strategy. It has been further extended and developed *via* taking nonlinearity, interactions, various types of mediating and outcome variables, as well as missing data into account in recent developments (e.g., Imai et al., 2010; Vanderweele and Vansteelandt, 2010; Pearl, 2012; Zhang and Wang, 2013).

Recently, mediation analysis has been applied to genetic association studies in which one can evaluate how genetic variants (e.g., single nucleotide polymorphisms (SNPs)) pass effects to mediators such as gene expression or DNA methylation (DNAm) to affect a disease risk (e.g., Liu et al., 2013; Huang et al., 2014; Huang et al., 2015). The genome-wide mediation analysis provides additional insight into the causal mechanisms of complex diseases. DNAm is an epigenetic phenomenon. Its status change reflects environmental exposures on the genome. DNAm can regulate gene expressions and can be potential biomarkers for the early prevention of stress-related disorders (Klengel et al., 2014). Properly maintained DNAms are necessary for regulating chromosomal stability and gene expressions. However, they can change the DNA activity when things go wrong, and lead to unexpected consequences. A growing body of literature shows that different environmental factors can alter the level of DNAm among individuals (e.g., Guida et al., 2015; Dongen et al., 2016). Abdolmaleky et al. (2004) showed that DNAm may modulate gene-environment interactions on psychiatry disorder. Li et al. (2003) reported that exposure to xenobiotics in early life can persistently change the pattern of DNAm, resulting in potentially adverse biological effects which may explain the increased risk in adulthood of some chronic diseases. All evidences demonstrate the important role of DNAm in mediating the effect of environmental exposures on disease outcomes. Successful identification of causal DNAm as potential biomarkers can offer novel insights into the early prevention of some diseases such as stress-related disorders.

In a typical DNAm study, the number of DNAm can be much larger than the number of sample size. Mediation analysis focusing on one mediator at a time is not efficient enough to handle thousands of mediators (e.g., CpG sites). Methods for multiple mediators have been proposed assuming different data distributions with different methods. Focusing on continuous mediators, Huang and Pan (2016) developed a testing procedure using Monte-Carlo resampling method to evaluate the statistical significance. However, it is time consuming when the computing resource is limited.

**Figure 1:**
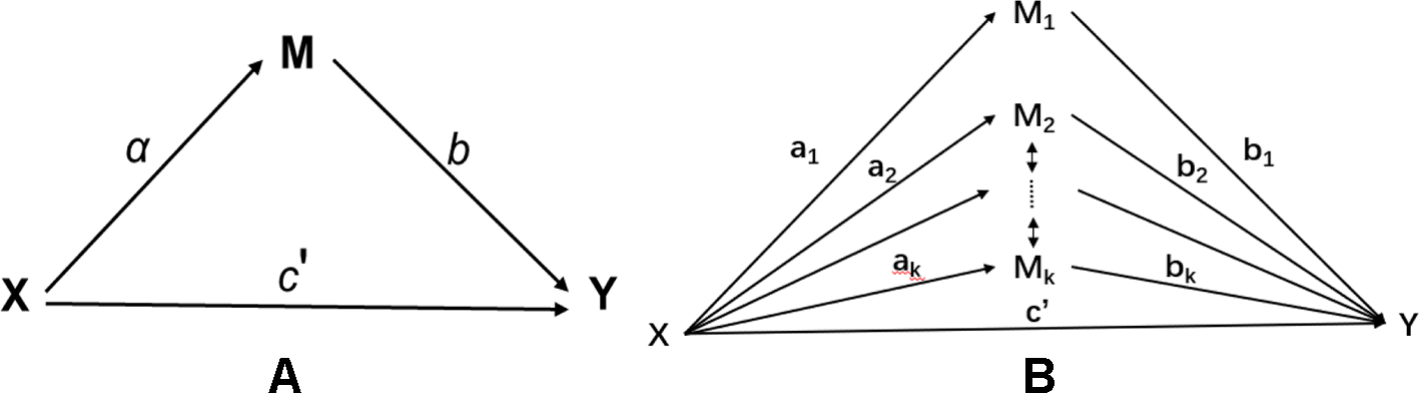
Mediation model: **(A)** single mediator model; **(B)** multiple mediator model with correlated mediators.

Let *X* be an exposure variable; *M_j_*, *j*=1,…,*k* be the *j*th mediator; and *Y* be an outcome variable. **Figure 1** illustrates the mediation model with a single mediator (a) and multiple mediators (b). In an epigenetic study, multiple mediators could be potentially correlated. For example, methylation signals in a given gene or region are typically correlated. Such correlation, if not properly handled, can lead to potential false positives or false negatives in traditional mediation analysis.

The high-dimensional and correlation nature of DNAm signatures (**Figure 1B**) motivates us to consider a high-dimensional mediation model, which is not a trivial extension of a low dimensional multiple mediator model studied in literature. Methodology development for mediation analysis with high-dimensional mediators is still in its infancy. Zhang et al. (2016) proposed a high-dimensional mediation analysis method. They first applied a sure independence screening (SIS) method to reduce the data dimension from ultra-high to high, then adopted a penalized regression to shrinkage coefficients of irrelevant variables to zero. After the shrinkage, those mediators with non-zero coefficients were refit in a low-dimensional regression model for further hypothesis testing. Such penalized regression methods typically produce biased estimators, especially when correlations between predictors exist. This method thus could face potential issues with either false positives or false negatives. Huang and Pan (2016) proposed to transform the correlated mediators into independent ones, then performed the mediation analysis on the transformed variables. Such a method solves the correlation issue but faces the difficulty of interpretation, since the transformed variable is a linear combination of the original mediators and does not have a direct interpretation.

High-dimensional data analysis is typically formulated with high-dimensional penalized regression models, with the purpose to select important features that can minimize the prediction error. Popular methods include LASSO (Tibshiranit, 1996), adaptive LASSO (Zou, 2006), and elastic net (Zou and Hastie, 2005). Although these methods can do variable estimation and selection simultaneously, they cannot quantify the estimation uncertainty. There has been a flourish of recent literature on testing low-dimensional coefficients in high-dimensional sparse regression models (e.g., Zhang and Zhang, 2014; Dezeure et al., 2015; Zhang and Cheng, 2017; Wang and Samworth, 2018). These methods essentially implement a debias technique, then perform hypothesis testing using the debiased estimators (Zhang and Zhang, 2014). Following the asymptotic normality, one can obtain a p-value or construct a confidence interval for each coefficient (Van de Geer et al., 2014). Taking the high dimensionality and correlation issue into account, in this article, we adopt a high-dimensional testing framework and conduct simultaneous inference under a high-dimensional sparse mediation model based on the recent de-sparsifying LASSO estimators (Zhang and Zhang, 2014). High-dimensional testing is embedded in the mediation model to handle the high dimensionality and correlation issues between mediators. We conduct extensive simulations to evaluate the performance of the methods and compare it with its counterpart. Application to two real data sets is given. Our method can be extended to other mediation analysis where high-dimensional mediators are observed.

## Statistical Method

**Figure 1A** demonstrates a single mediation model. There are two types of effect from *X* to *Y*: (1) the direct effect from *X* to *Y*, denoted as $c^{\prime }$; and (2) the indirect effect from *X* to *Y via* the intermediate mediation variable *M*. The indirect effect measures the amount of mediation which comes from two sources: i) the effect from *X* to *M*, denoted as *a*; and ii) the effect from *M* to *Y*, denoted as *b*. The product of *a* and *b* defines the indirect effect. The total effect *c* from *X* to *Y* contains two parts, i.e., $c=c^{\prime }+ab$. By fitting three different regression models, one can use the Sobel’s method (Sobel, 1982) to estimate the standard error of $\hat{a}\hat{b}$ from which the significance of mediation effect can be assessed.

The single mediator model shown in **Figure 1A** can be extended to a multiple mediator model by fitting a multiple regression model involving both the exposure and the mediator variables. The multiple mediator model is given as follows,

(1) $$\begin{array}{l} Y=\theta_{1}+cX+e_{1}\\ M_{j}={\theta^{\prime }}_{j}+a_{j}X+\varepsilon_{j},j=1,...,k,\\ Y=\theta_{2}+c^{\prime }X+\sum_{j=1}^{k}b_{j}M_{j}+e_{2}, \end{array}$$

where *M_j_*, *j*=1,..,*k* is the *j*th mediator variable; *c* represents the total effect from the independent variable *X* to the dependent variable *Y*; $c^{\prime }$ represents the direct effect from *X* to *Y* adjusting for the effects of multiple mediators; the indirect effect from X to Y mediated by *M_j_* is denoted by *a_j_b_j_*. The total mediation effect can be obtained as $c-c^{\prime }$ or $\sum_{j=1}^{k}a_{j}b_{j}$. When the response variable *Y* is a categorical variable, method to estimate the total mediation effect based on the product measure, *a_j_b_j_*, is less susceptible to the scaling problem since only the *b_j_* coefficient is from a categorical regression analysis (MacKinnon, 2008). Model (1) is for continuous *Y* variable. For a categorical response, Model (1) becomes,

(2) $$\begin{array}{l} E(Y)=\theta_{1}+cX,\\ M_{j}={\theta^{\prime }}_{j}+a_{j}X+\varepsilon_{j},j=1,...,k,\\ E(Y)=\theta_{2}+c^{\prime }X+\sum_{j=1}^{k}b_{j}M_{j}. \end{array}$$

As we mentioned in the *Introduction* section, a genomic mediation study often involves high-dimensional mediators. In many cases, the number of mediators is far beyond the sample size (*k*>>*n*). For example, the number of DNAm loci can be nearly half million, far more than the sample size. Another phenomenon for genomic mediators is that they are often correlated. Both the curse of dimensionality and correlation between mediators cause estimation problems in Model (1) and (2). Classical regression analysis cannot be directly adopted to deal with the estimation and testing problem appeared in the third equation in Model (1) and (2). To solve both the high dimensionality and correlation problem, we propose to adopt a high-dimensional testing framework which is focused on de-sparsified LASSO estimators (Zhang and Zhang, 2014). The detailed estimation and testing procedure for the proposed high-dimensional mediation testing framework is given as follows:

**Step 1**: First apply an SIS procedure to reduce the methylation dimension from ultra-high to high dimension (Fan and Lv, 2008). According to the SIS algorithm, the top *d*=*n*/*log*(*n*) methylation variables with the largest effects were remained in the model when the response *Y* is a continuous variable. For a binary response, the top *d*=*n*/*log*(*n*) variables can be kept in the model. SIS theoretically guarantees that no true signals are removed from the model. The SIS step can be based on the third or the second regression equation in Model (2). For a binary response *Y*, Zhang et al. (2016) suggested that SIS can be done based on the second equation in Model (2). For a continuous response variable, the SIS step can be done based on the third regression equation in (2). After SIS, the number of methylation loci is reduced from *k* to *d*. We then focused our analysis to these *d* methylation variables to test mediation effects. Denote the remaining methylation loci after the SIS step as *M_j_*,*j*=1,…,*d*.

**Step 2**: In the second step, we fit the following model,

(3) $$E\left(Y\right)=\theta_{2}+c^{\prime }X+\sum_{j=1}^{d}b_{j}M_{j}$$

Other covariates can also be fitted to this model. Since the dimension *d* can still be relatively large after the SIS step, regular least squares estimation will not work well. For high-dimensional data, penalized regressions are commonly applied for simultaneous variable selection and estimation. However, penalized estimators are biased and cannot be directly used for testing or confidence interval construction. Zhang and Zhang (2014) first time proposed a de-biased estimator for high-dimensional data. Let ${\hat{b}}_{lasso}$ be the LASSO estimators. For a continuous response variable *Y*, A de-biased estimator, also called a de-sparsified estimator, is a bias-corrected estimator which can be given as,

(4) $${\hat{b}}_{j}=\frac{Z_{j}^{T}Y}{Z_{j}^{T}M_{j}}-\sum j\neq l\frac{Z_{j}^{T}M_{l}}{Z_{j}^{T}M_{j}}{\hat{b}}_{lasso,l}$$

where ${\hat{b}}_{j}$ is the bias-corrected coefficient of the *j*th methylation *M_j_*; ${\hat{b}}_{lasso,l}$ is the coefficient of the *l*th *M_l_* estimated by fitting a LASSO regression; *Z_j_* is the regularized residuals obtained by $Z_{j}=M_{j}-M_{-j}{\hat{\gamma }}_{lasso}$, where ${\hat{\gamma }}_{lasso}$ is the regression coefficients obtained based on a LASSO regression by regressing *M_j_* on all other *M* except the *j*th *M_j_* denoted as $M_{-j}$ . Van de Geer et al. (2014) proved the asymptotic normality of the de-sparsified estimate, i.e.,

$$u_{j}=\frac{\sqrt{n}\left({\hat{b}}_{j}-\gamma_{j}^{0}\right)}{\sigma_{\int }\sqrt{\Omega_{jj}}}\overset{d}{\to }N\left(0,1\right)\;\;\;as\;\;\;p\geq n\;\to \infty$$

where $\gamma_{j}^{0}$ represents the true regression coefficient; $\sigma_{\epsilon }$ can be calculated by using the scaled LASSO algorithm (Sun and Zhang, 2012), and Ω*_jj_* can be calculated by,

$${\text{Ω}}_{jj}=\frac{nZ_{j}^{T}Z_{j}}{\left[Z_{j}^{T}Z_{j}\right]\left[Z_{j}^{T}Z_{j}\right]}$$

Under the null that $H_{0}:\gamma_{j}^{0}=0$, we can get *p*-values for all the *d* methylation loci based on the asymptotic normality (Van De Geer et al., 2014).

For a binary response, Van de Geer et al. (2014) also proved the asymptotic normality for the de-sparsified estimates. Let $W={\left(X,M\right)}^{T},\;\beta ={\left(c^{\prime },b\right)}^{T}$ , and $L_{\beta }\left(y,W\right)=L\left(y,W\beta \right)$ be a loss function, and define ${\dot{L}}_{\beta }=\frac{\partial }{\partial \beta }L_{\beta }$ and ${\overset{..}{L}}_{\beta }=\frac{\partial^{2}}{\partial \beta \partial \beta^{T}}L_{\beta }$ , and further define $\varphi_{L}:=\overset{n}{\underset{i=1}{\sum }}L\left(y_{i},w_{i}\beta \right)/n$.≔ The LASSO estimator for the mediation coefficients **β** is given as $\hat{\beta }=\underset{\beta \;}{\arg\min}\left(\varphi_{L}+\lambda \forall ||\beta |{|}_{1}\right),$ where λ is a tuning parameter. Define $\hat{\Sigma }\text{:=}\varphi_{{\overset{..}{L}}_{\hat{\beta }}}$ and construct $\hat{\Theta }={\hat{\Theta }}_{LASSO}$ by doing a nodewise LASSO with $\hat{\Sigma }$ as input. Then the de-sparsified LASSO estimator is given as $\tilde{\beta }:=\hat{\beta \;}-\hat{\Theta }\varphi_{{\dot{L}}_{\hat{\beta }}}$. van de Geer et al. (2014) provided a detailed algorithm for computing the de-sparsified LASSO estimators in a generalized linear model framework. They also proved the asymptotic normality of the de-sparsified estimate, i.e.,

$$u_{j}=\frac{\sqrt{n}\left({\tilde{\beta }}_{j}-\gamma_{j}^{0}\right)}{{\hat{\sigma }}_{j}}\overset{d}{\to }N\left(0,1\right)\;\;\;as\;\;\;p\geq n\;\to \infty$$

where ${\hat{\sigma }}_{j}^{2}={\left(\hat{\Theta }P_{{\dot{L}}_{\hat{\beta }}{\dot{L}}_{\hat{\beta }}^{T}}\;{\hat{\Theta }}^{T}\right)}_{j,j}$. Similarly, we can get a p-value for each mediator based on the asymptotic normality property.

Let the p-values for all the *d* methylation loci denoted as *P_b_*=(*P*_1,_*_b_*,*P*_2,_*_b_*,…,*P_d,b_*) where *P_j,b_* can be calculated as $P_{j,b}=2\left\{1-\Phi \left(\frac{\sqrt{n}{\hat{b}}_{j}}{\sigma_{\epsilon }\sqrt{{\text{Ω}}_{jj}}}\right)\right\}$ for a continuous *Y* or as $P_{j,b}=2\left\{1-\Phi \left(\frac{\sqrt{n}{\hat{b}}_{j}}{{\hat{\sigma }}_{j}}\right)\right\}$ for a discrete *Y*.

**Step 3**: Let *S*={*t*:*P_t,b_*< 0.05}, which is based on the high-dimensional inference in the second step. For testing *H*_0_:*a_t_* = 0, we denote the testing p-value as *P_t,a_*

$$P_{t,a}=2\left\{1-\Phi \left(\frac{\left|{\hat{a}}_{t}\right|}{{\hat{\sigma }}_{t}}\right)\right\},$$

where t∈S, ${\hat{a}}_{t}$ is the ordinary least squares estimator for *a_t_* and ${\hat{\sigma }}_{t}$ is the corresponding estimated standard error, by fitting the 2nd regression equation in Model (2).

**Step 4**: We reject the null hypothesis of no mediation effect for *M_t_* only if both *a_t_* and *b_t_* are significant. The p-value for the joint significance test is defined as,

$$P_{t}^{*}=\max\left(P_{t,a},P_{t,b}\right)$$

A methylation locus has a significant mediation effect if $P_{t}^{*}<0.05$. This is also a so called intersection-union test (Berger and Hsu, 1996).

**Remark 1**: To make the paper self-contained, here we briefly introduce the High-dimensional mediation analysis (HIMA) method proposed by Zhang et al. (2016). The HIMA method involves three major steps:

Step 1: (Screening) Use the SIS (Fan and Lv, 2008) to identify a subset of top mediators.

Step 2. (MCP-penalized estimate). Apply the MCP-based penalized regression to do simultaneous variable selection and estimation based on the variables from step 1.

Step 3. (Joint significance test). For those mediators with non-zero coefficients from step 2, fit a regression model again and get a p-value for testing each coefficient, then, taking the maximum of this p-value and the p-value for testing the α effect as the final p-value to assess the significance of the mediation effect.

**Remark 2**: Our method has two advantages: 1) It fits multiple mediators in one regression model and do the testing, rather than fitting and testing mediation effect one at a time. Statistically speaking, this yields more robust and efficient estimation and testing results; and 2) Different from Zhang et al. (2016), our method is a simultaneous inference in a high-dimensional sparse regression model implemented with a de-biasing technique. The de-sparsifying strategy can well handle correlations between methylation loci, as demonstrated in the simulation study.

## Simulation Studies

We conduct extensive simulations to evaluate the performance of the proposed method and compare it with the HIMA method proposed by Zhang et al. (2016). In the follows, we denote our method as HDMA (high dimensional mediation analysis) and the method by Zhang et al. (2016) as HIMA. Data are generated following Model (2), where the exposure variable *X* is generated from a binomial distribution, i.e., *B*(*n*,0.74) in which the probability 0.74 is determined based on the proportion of drinking in the first real set (see the real data analysis section for details). To have a fair comparison, we follow the simulation setup for the regression coefficients as given in Zhang et al. (2016). The first 8 elements of *b*(*b_j_,j* = 1,…,8) are given as (0.8,0.7,0.6,0.5,0,0,0.5,0.5)*^T^*, and the first 8 elements of *a*(*a_j_*,*j* = 1,…,8)are given as (0.35,0.25,0.35,0.55,0.55,0.55,0,0)*^T^*. The rest of *as* and $b^{\prime }s$ are all set to zero. Under this setting, the first four methylation loci have significant mediation effects while the rest have no effect.

For the intercept terms, we set *θ*_2_ = – 4.5 and $\theta_{j}^{'}=1$. We also consider different correlations among the mediators, i.e., *ρ* = 0, and 0.8. When the direct effect $c^{\prime }=0$, the model is a complete mediation model in which exposures affect outcome only through mediators. In this case, the total effect $c=c^{\prime }+\sum_{j=1}^{k}a_{j}b_{j}=0.94$. When the direct effect $c^{\prime }>0$, the model is a partial mediation model. For the partial mediation model, we set $c^{\prime }=0.5$ and the total effect $c=c^{\prime }+\sum_{j=1}^{k}a_{j}b_{j}=1.44$.

We simulate *k* methylation loci which follow a multivariate normal distribution, i.e., $M_{i}\sim MVN\left(1+a_{i}X_{i},\Sigma \right)$ , where $a_{i}={\left(0.35,\;0.25,\;0.35,\;0.55,\;0.55,\;0.55,\underset{k-6}{\underset{︸}{0,...,0}}\right)}^{T}$ and $\Sigma_{st}=\rho^{\left|s-t\right|}.$ Then we sample the response *Y_i_*∼*Ber*(1,*p_i_*), where $p_{i}=\text{exp}(\eta_{i})/\left(1+\text{exp}\left(\eta_{i}\right)\right)$ and $\eta_{i}=-4.5+c^{\prime }X_{i}+\sum_{j=1}^{k}b_{j}M_{ij}$.

We evaluate the performance of our method (HDMA) in terms of false positive rate and power and compare with HIMA. We report the power (*M_1_*∼*M*_4_) and the type I error (*M_5_*∼*M*_8_) for each locus. For the rest of the *k*-8 loci, we report the averaged type I error rate. All simulations are based on 1000 replications under different sample sizes, i.e., *n* = 300 and 600 and different correlations, i.e., *ρ* = 0 and 0.8.

**Table 1** lists the results for binary responses assuming a complete mediation effect, i.e., $c^{\prime }=0$. There are several observations: (i) HIMA and HDMA have very similar power and size when there are no correlations between *M* (ρ = 0) under different scenarios. However, HDMA has substantially higher power than HIMA does when ρ = 0.8; (ii) The testing power decreases as the data dimension increases for both methods. For example, the power of testing M_1_ is 0.754 for HDMA with k = 100, but decreases to 0.721 with k = 5000, when fixing n = 300 and ρ = 0; (iii) The power increases as the sample size increases. For example, when fixing ρ = 0.8 and k = 1000, the power increases from 0.598 to 0.951 for testing M_1_ when the sample size increases from 300 to 600, a 59% increase; and (iv) HDMA is not sensitive to the correlation structures while HIMA suffers significantly from power loss when there are high correlations between the M variables. The difference is even more striking when the sample size increases from 300 to 600. For example, the power difference for testing M_1_ is 0.014 for HDMA compared to 0.238 for HIMA when ρ is increased from 0 to 0.8, when fixing n = 600 and k = 1000. Similar patterns were observed for the other three M variables.

**Table 1:** List of the power and type I error rate under different sample sizes and correlations with data analyzed with HDMA and HIMA.

*n*	*k*		Method	*M*_1_	*M*_2_	*M*_3_	*M*_4_	*M*_5_	*M*_6_	*M*_7_	*M*_8_	*M*_other_
300	100	0	HIMA	0.754	0.467	0.723	0.849	0.025	0.022	0.034	0.047	0.001
			HDMA	0.754	0.460	0.713	0.825	0.021	0.017	0.034	0.046	0.001
		0.8	HIMA	0.502	0.241	0.362	0.377	0.075	0.070	0.028	0.019	0.001
			HDMA	0.649	0.348	0.445	0.422	0.062	0.062	0.023	0.012	0.000
	1000	0	HIMA	0.763	0.478	0.653	0.702	0.008	0.008	0.049	0.029	0.001
			HDMA	0.763	0.476	0.660	0.697	0.008	0.006	0.044	0.032	0.000
		0.8	HIMA	0.513	0.194	0.370	0.386	0.078	0.072	0.013	0.023	0.000
			HDMA	0.598	0.297	0.399	0.417	0.060	0.055	0.012	0.019	0.000
	5000	0	HIMA	0.714	0.437	0.590	0.528	0.003	0.002	0.024	0.029	0.000
			HDMA	0.721	0.440	0.589	0.549	0.002	0.002	0.027	0.027	0.000
		0.8	HIMA	0.545	0.182	0.374	0.386	0.081	0.076	0.024	0.017	0.000
			HDMA	0.577	0.267	0.413	0.388	0.047	0.045	0.017	0.013	0.000
600	100	0	HIMA	0.957	0.769	0.969	0.990	0.008	0.010	0.046	0.051	0.001
			HDMA	0.957	0.769	0.969	0.996	0.019	0.015	0.046	0.052	0.001
		0.8	HIMA	0.776	0.352	0.505	0.476	0.044	0.047	0.027	0.018	0.001
			HDMA	0.950	0.686	0.781	0.602	0.069	0.059	0.022	0.021	0.001
	1000	0	HIMA	0.965	0.770	0.967	0.979	0.004	0.004	0.039	0.043	0.000
			HDMA	0.965	0.770	0.966	0.977	0.013	0.008	0.040	0.043	0.001
		0.8	HIMA	0.727	0.366	0.494	0.443	0.052	0.046	0.037	0.015	0.000
			HDMA	0.951	0.685	0.790	0.632	0.060	0.071	0.026	0.021	0.000
	5000	0	HIMA	0.962	0.760	0.959	0.945	0.005	0.007	0.057	0.054	0.000
			HDMA	0.963	0.761	0.960	0.941	0.005	0.007	0.054	0.054	0.000
		0.8	HIMA	0.733	0.391	0.503	0.472	0.068	0.058	0.041	0.025	0.000
			HDMA	0.924	0.666	0.759	0.604	0.070	0.067	0.039	0.024	0.000

**Figure 2:**
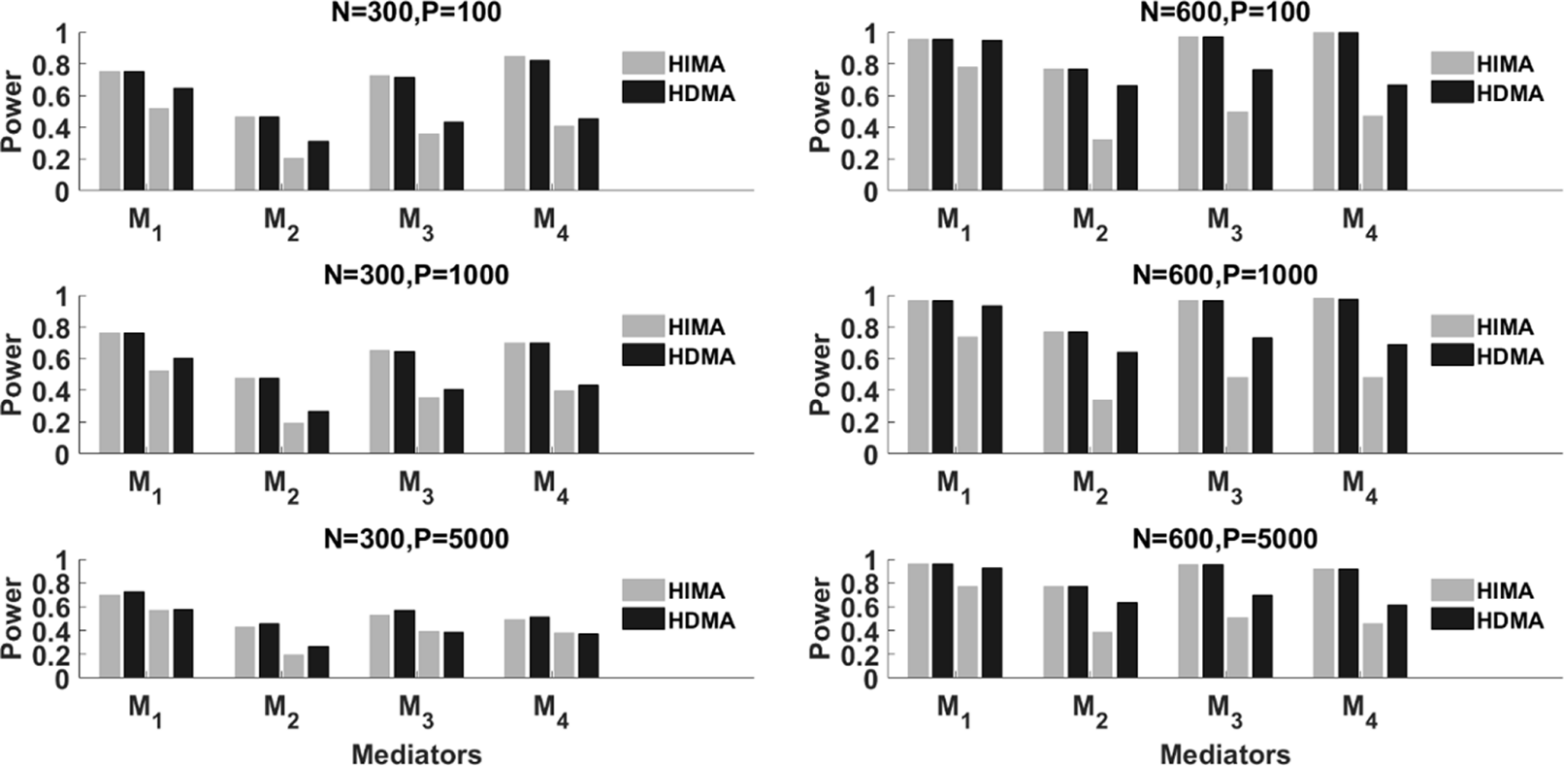
The power of HIMA (light gray) and HDMA (black) under different sample sizes, data dimensions, and correlations. *M*_1_∼*M*_4_ refer to the first four significant mediators. There are four power bars corresponding to each mediator. The left and right two bars correspond to the case with correlation *ρ*=0 and 0.8 respectively.

**Figure 2** summarizes the results with partial mediation, i.e., $c^{\prime }=0.5$. We consider N = 300 and 600, p = 100, 1000 and 5000, and ρ = 0 and 0.8. Corresponding to each mediator, there are four power bars. The left two correspond to the case with correlation ρ = 0, while the right two correspond to the case with ρ = 0.8. For a fixed sample size, the power typically decreases as the data dimension (p) increases. This is because of the increase of the noise features. When ρ = 0 (the independent case), HIMA and HDMA perform very similarly under different scenarios. However, when the correlation increases to ρ = 0.8, we observe a power gain by HDMA compared to HIMA under a sample size of 300. As the sample size increases from 300 to 600, we observe substantial power gain for HDMA. This shows the advantage of HDMA which can take care of the high correlation structure among the mediators.

**Figure 3:**
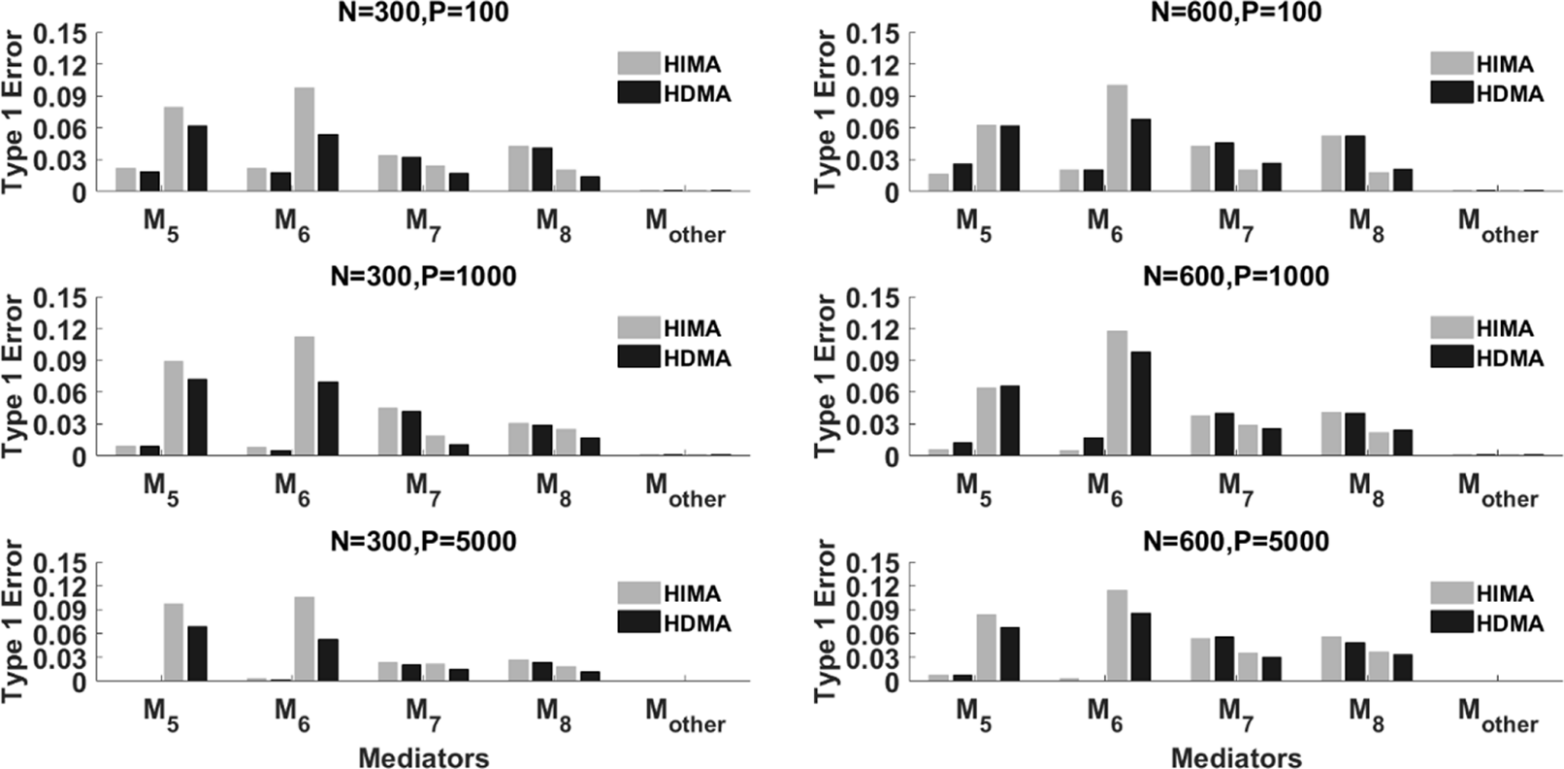
The type I error of HIMA (light gray) and HDMA (black) under different sample sizes, data dimensions, and correlations. *M*_1_ and *M*_4_ refer to the first four significant mediators. There are four power bars corresponding to each mediator. The left and right two bars correspond to the case with correlation *ρ*=0 and 0.8 respectively.

**Figure 3** displays the type I error rate of the two methods. M*_other_* represents all p-8 zero effect mediators. The type I error for M*_other_* is calculated as the average type I error of the p-8 mediators. Again, each mediator has four bars. The left two correspond to ρ=0 while the right two correspond to ρ=0.8. Overall, the type I errors for the two methods are reasonably controlled, especially under a large sample size (N = 600). When the correlation is high, i.e., ρ=0.8, for some mediators such as M_5_ and M_6_, HIMA has a higher false positive rate than HDMA does. This indicates the advantage of HDMA in false positive control when there are high correlations among mediators.

In summary, HDMA shows relative advantages over HIMA under different scenarios, especially when there are high correlations among mediators. As correlations are highly expected in real methylation data, HDMA can be an alternative strategy to HIMA and is generally safe to apply.

## Real Data Analysis

We apply the HDMA method to two real data sets with methylation loci as the mediators. DNAms play key roles in regulating many cellular processes and are associated with human diseases (Robertson 2005). The first data set involves DNAm mediating the effect of alcohol consumption on epithelial ovarian cancer (EOC) status. Alcohol may induce DNAm alterations, which could trigger alcohol-induced carcinogenesis (Varela-Rey et al., 2013). In the second data set, we evaluate the effect of childhood maltreatment on post-traumatic stress disorder (PTSD) in adulthood, mediated by DNAms. It is hypothesized that childhood maltreatment affects biological processes *via* DNAm, which can have negative consequences late in life (e.g., Mehta et al., 2013; Klengel et al., 2016).

### Case Study 1: Mediation Analysis of Alcohol Consumption, DNam, and EOC Status

The participants with age ranging from 27 to 91 were recruited between the year 1999 and year 2007 in the Mayo Clinic Ovarian Cancer. They were women of European ancestry who were invasive EOC cases and controls one-to-one matched on the basis of age (within 1-year). After eliminating missing values and other quality control, 196 cases and 202 controls were retained for further analysis. The exposure variable is alcohol consumption. Information on alcohol use was obtained *via* a written questionnaire asking “Do you currently drink alcoholic beverages?” DNAms are the mediators and EOC status is the outcome. We would like to identify the mediators and further quantify the mediation effect. Readers are referred to Koestler et al. (2014) and Wu et al. (2018) for more details about the data.

**Table 2** summarizes the lifestyle and demographic characteristics of the study population. The Student *t*-test or Chi-square test is used for comparisons between groups for continuous or categorical variables, respectively. As can be seen in the table, alcohol consumption is significantly lower in cases compared to controls. Enrollment year shows a significant difference in proportions between cases and controls. Thus, we include the enrollment year as a covariate in further mediation analysis.

**Table 2:** Partial list of covariates and their association with case/control status.

	Case (N = 196)	Control (N = 202)	Total (N = 398)	p value
**Age at diagnosis/interview**				
Mean(SD)	62.31 (12.36)	62.37 (12.69)	62.34 (12.51)	0.965
**Enrollment year**				
1999–2002 year	76 (38.78%)	91 (45.05%)	167 (41.96%)	<0.001
2003 year	17 (8.67%)	27 (13.37%)	44 (11.06%)	
2004 year	25 (12.76%)	42 (20.79%)	67 (16.83%)	
2005 year	30 (15.31%)	17 (8.42%)	47 (11.81%)	
2006–2007 year	48 (24.49%)	25 (12.38%)	73 (18.34%)	
**Alcohol use at study enrollment**				
Yes	123 (62.76%)	172 (85.15%)	295 (74.1%)	<0.001
No	73 (37.24%)	30 (14.85%)	103 (25.9%)	
**Minnesota (MN) state**				
Other	93 (47.45%)	82 (40.59%)	175 (43.97%)	0.202
MN	103 (52.55%)	120 (59.41%)	223 (56.03%)	
**Smoking at study enrollment**				
No	178 (90.82%)	192 (95.05%)	370 (92.96%)	0.145
Yes	18 (9.18%)	10 (4.95%)	28 (7.04%)	

Leukocyte-derived DNA was assayed with the Illumina Infinium HumanMethylation27 Beadchip platform and underwent quality control procedures at the Mayo Clinic Molecular Genome Facility (Koestler et al., 2014). The methylation beta values (*β*) of each CpG locus was logit-transformed (log(*β/*(1-*β*))) to get the M-value for further analysis. A total of 25,926 CpG sites were remained for analysis after normalization and adjusting for any batch or plate effects. Study shows that heterogeneity in white blood cells has the potential to confound DNAm measurements and statistical treatment is needed to correct for this confounding effect (Adalsteinsson et al., 2012). Similarly, variation in cell-type proportions across samples has the potential to confound the mediation effect of DNAm on the association of alcohol consumption and EOC status (Titus et al., 2017). We thus include the predicted proportions of the leukocyte sub-types for each of the study samples as covariates in the analysis, following a mixture deconvolution method by Houseman et al. (2012).

Since the response is a binary variable, we apply a logistic regression for the first and third regression equation in Model (2), while including enrollment year as a covariate. Note that the cell type data should be included whenever methylation signals are included in the model. Including the enrollment year (Enroll) and the proportion of cell type (CellType), Model (2) becomes,

$$\begin{array}{l} \text{logit}(P\text{)=}\theta_{1}+c_{Alcohol}\text{Alcohol+}\lambda_{1}^{T}\text{Enroll}\\ {\text{CpG}}_{j}={\theta^{\prime }}_{j}+a_{j}\text{Alcohol+}\lambda_{2}^{T}\text{Enroll+}\delta_{1}^{T}\text{CellType+}\varepsilon j.j=1,\ldots ,k,\\ \text{logit(}P\text{)=}\theta_{2}+{c^{\prime }}_{Alcohol}\text{Alcohol}+\sum_{j=1}^{k}b_{j}CpG_{j}+\lambda_{3}^{T}\text{Enroll}+\delta_{2}^{T}\text{CellType} \end{array}$$

The coefficient estimates for the total effect is given as ${\hat{c}}_{Alcohol}$=-1.310 (p-value < 0.001), indicating a significant protective effect of alcohol consumption on EOC status.

**Figure 4:**
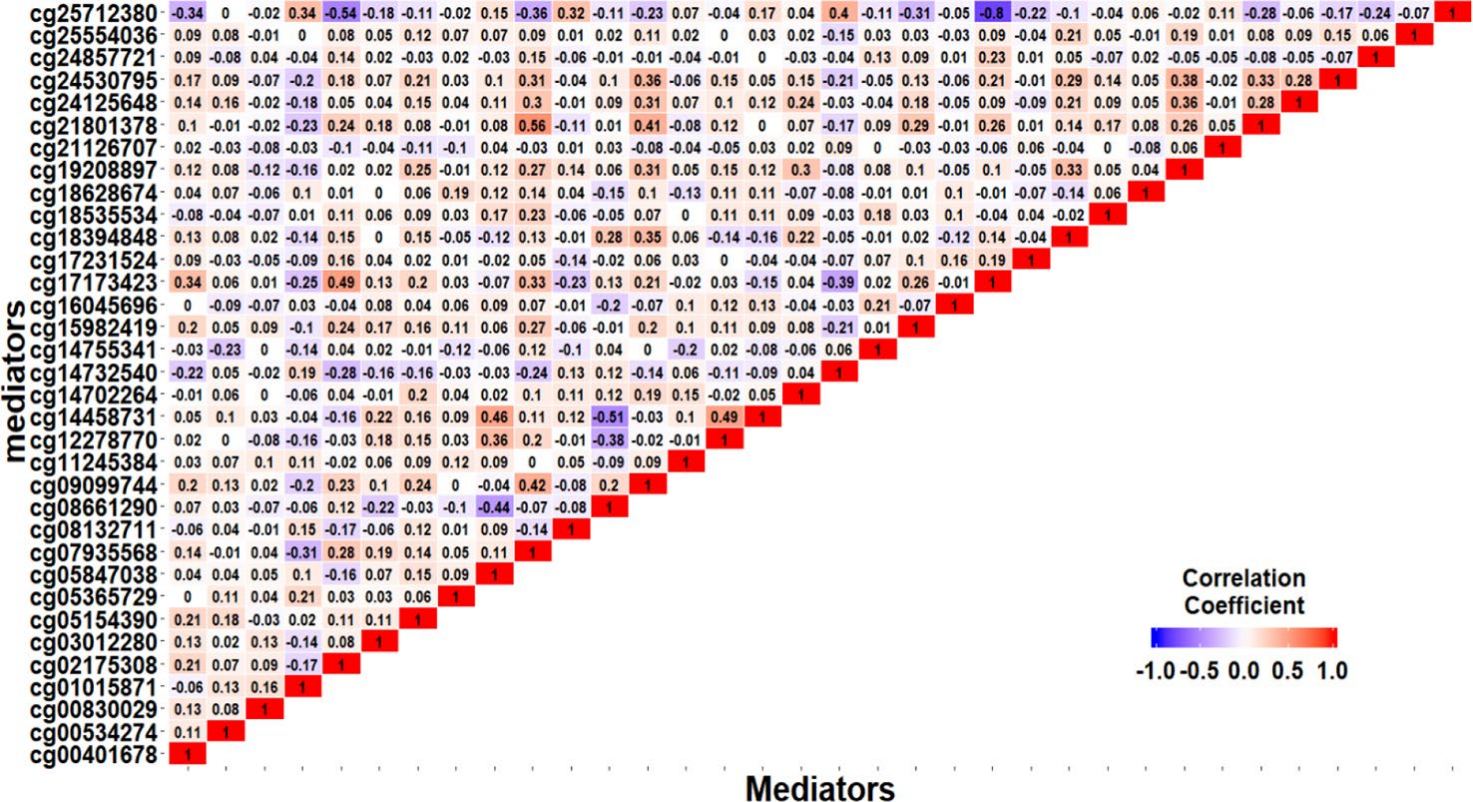
The correlation structure among the 34 CpG sites.

We apply the SIS algorithm to reduce the methylation dimension to 34 (*n*/2log(*n*)), then apply the HDMA and HIMA methods for further inference. **Table 3** lists the findings by the two methods. Our method identified four CpGs with important mediation effects while HIMA identified two CpGs. Two CpGs, namely cg12278770 and cg03012280, overlap in two methods. A heatmap in **Figure 4** shows that there are moderate correlations among the 34 CpG sites. Thus, it is not surprising to see that HDMA identifies more CpG mediators than HIMA does.

**Table 3:** List of significant CpGs identified by HDMA and HIMA.

Method	CpG	Chr	Gene name	$\hat{a}$	$\hat{b}$	$\hat{a}\hat{b}$	% of total effect	*p*-value
HDMA	cg18394848	12	*K-RAS*	−0.076	1.772	−0.136	10.343	0.008
	cg08132711	4	*KSP37*	−0.080	1.207	−0.096	7.313	0.033
	cg12278770	1	*FAM167B*	−0.071	−2.045	0.144	11.012	0.005
	cg03012280	15	*ZFYVE19*	−0.175	−0.878	0.153	11.709	0.002
HIMA	cg12278770	1	*FAM167B*	−0.071	−0.828	0.058	4.461	0.002
	cg03012280	15	*ZFYVE19*	−0.175	−0.525	0.092	7.010	0.004

**Figure 5:**
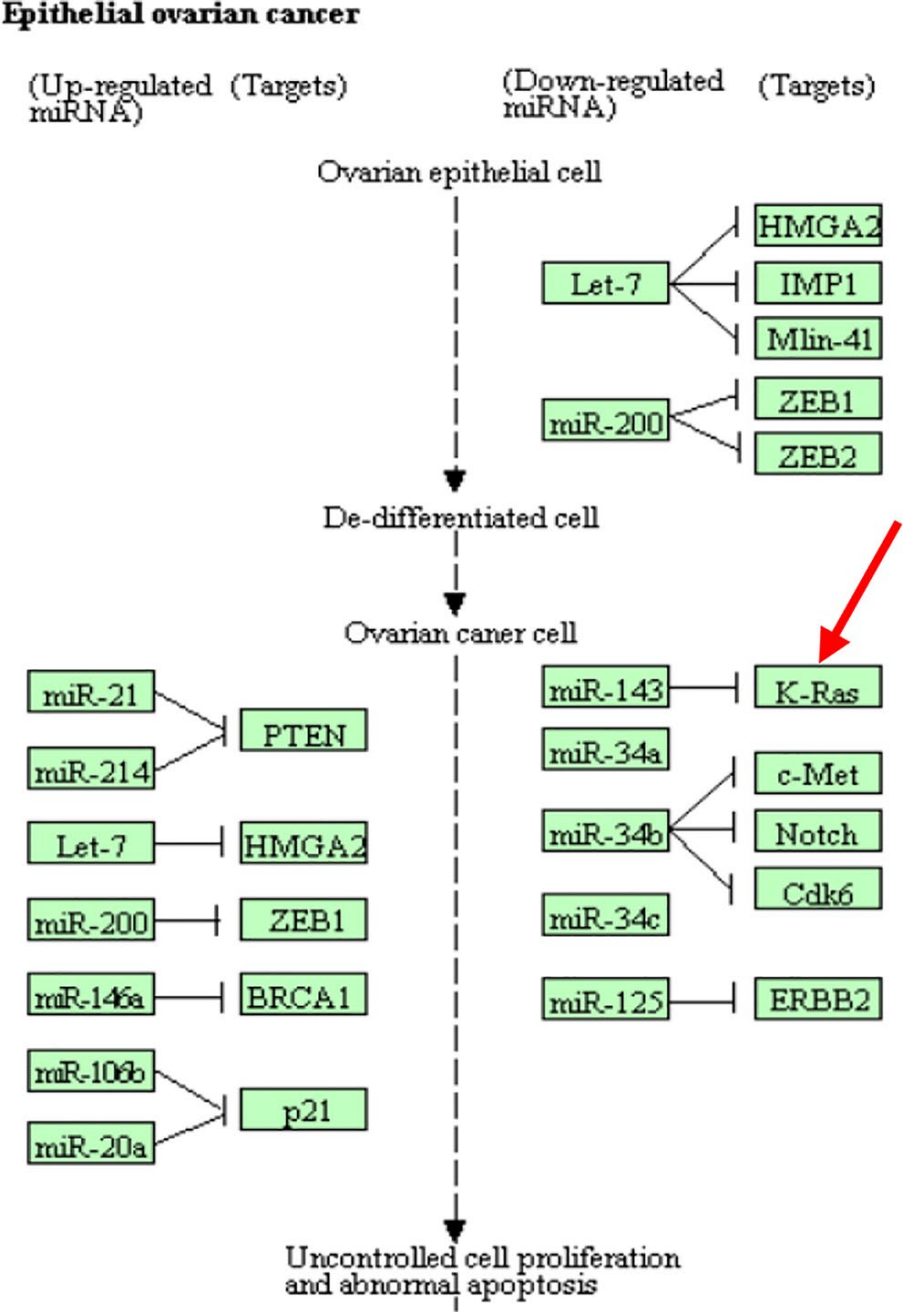
Partial EOC pathway extracted from the KEGG database (https://www.kegg.jp/kegg-bin/show_pathway?hsa05206).

CpG site cg18394848 resides in gene *K-RAS*. Nakayama et al. (2008) examined the *K-RAS* mutations in relation to extracellular signal-regulated protein kinase (*ERK*) activation in 58 ovarian carcinomas. Auner et al. (2009) drew a conclusion that *K-RAS* mutation is a common event in ovarian cancer primarily in carcinomas of lower grade, lower FIGO stage, and mucinous histotype. KEGG pathway shows that this gene is involved in the pathogenesis of ovarian cancer (**Figure 5**). This evidence indicates that cg18394848 could be an important epigenetic marker which mediates the effect of alcohol consumption on EOC pathogenesis.

**Figure 6:**
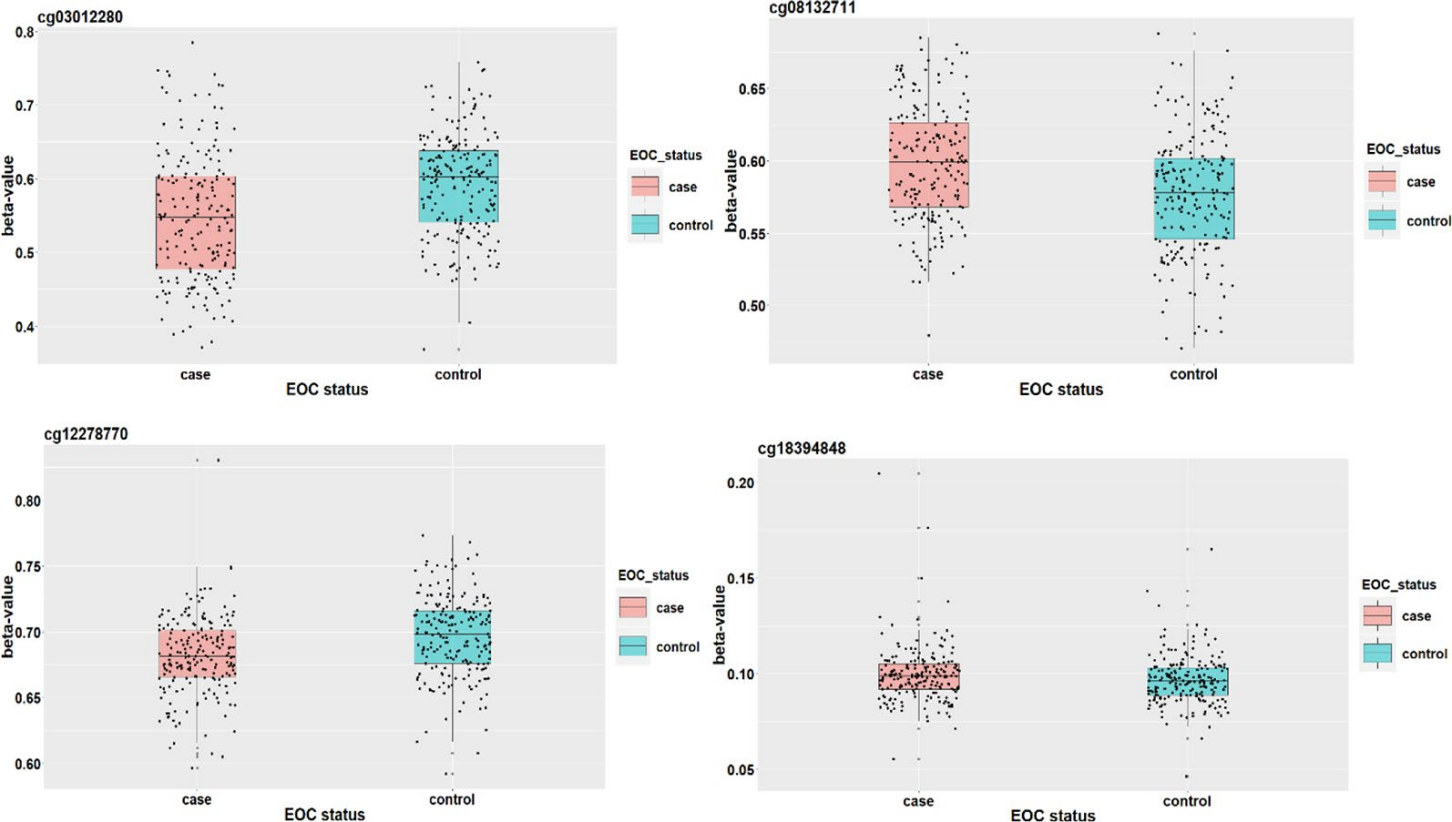
The DNAm status (*β* value) of cg18394848, cg08132711, cg12278770, and cg03012280 between EOC cases and controls.

Elgaaen et al. (2010) found that gene *KSP37* correlates strongly with histology, stage, and outcome in ovarian carcinomas. Thus, cg08132711 (in gene *KSP37*) can also be a potential epigenetic marker associated with the EOC status. Although we do not find direct literature support about the two genes *FAM167B* and *ZFYVE19* where cg12278770 and cg03012280 are respectively located in, a two samples t-test results show that there are significant differences on methylation signals of cg12278770 and cg03012280 between cases and controls. The *t*-test statistics (p-value) are *t_cg_*_12278770_=4.881(*P*<0.001) and *t_cg_*_0301220_=5.415(*P*<0.001). It suggests that these two CpG sites may act as important players to mediate the effect of alcohol intake on EOC status (**Figure 6**).

### Case Study 2: Mediation Analysis of Childhood Maltreatment, Dnam, and PTSD

The data came from the Grady Trauma Project study recruiting Afro-American participants from Atlanta inner-city residents, approved by the Institutional Review Board of Emory University School of Medicine and Grady Memorial Hospital (Wingo et al., 2018). A growing body of literature indicates that DNAm plays pivotal roles in the disease process of PTSD and in vulnerability and resilience to PTSD (Uddin et al., 2011; Lutz and Turecki, 2014). Studies also show that childhood maltreatment is associated with DNAm changes of multiple loci in adulthood (Mehta et al., 2013). We apply the proposed method to establish the link between childhood maltreatment and PTSD and further evaluate the mediating role of DNAm. The data set contains baseline information, cell composition, and DNAm. We adopt the modified PTSD Symptom Scale (PSS) and the Beck Depression Inventory (BDI) to classify cases and controls. Cases with current symptoms of comorbid PTSD and depression are defined as having a PSS score ≥14 and a BDI score ≥14. Controls are defined as having neither PTSD nor depressive symptoms, as mirrored by a PSS score ≤7 and BDI score ≤7, despite being exposed to trauma (Beck et al., 1961; Foa et al., 2000; Wingo et al., 2018). We eliminate observations with missing values and exclude those with PTSD treated since the treatment might affect DNAm changes which can complicate the mediation effect. Finally, 54 controls and 74 cases are retained for further analysis.

**Table 4** summarizes the demographic characteristics of the study population. Ranges of age in case and control are (27.97, 57.97) and (30.69, 56.79), respectively. There is no statistical significance among the selected variables such as age, sex, and body mass index (BMI), but childhood sexual/physical abuse moderate to extreme is significantly higher for cases compared to controls. The same analysis plan as detailed in Case Study 1 is applied here. Since no clinical factors show statistical significance, we do not include any covariates in our mediation model. Next, we apply HDMA and HIMA to test which DNAm plays a mediating role between childhood maltreatment and PTSD.

**Table 4:** Partial list of covariates and their association with PTSD case/control status.

Variables	Case (N = 74)	Control (N = 54)	Total (N = 128)	p-value
**Age**				
Mean (SD)	40.97 (13.00)	43.74 (13.05)	42.141 (13.04)	0.238
**Sex**				
Male	52 (70.27%)	36 (66.67%)	88 (68.75)	0.771
Female	22 (29.73%)	18 (33.33%)	40 (31.25)	
**BMI**				
Mean (SD)	31.433 (7.82)	31.614 (8.10)	31.510 (7.91)	0.899
**Childhood sexual/physical abuse moderate to extreme**				
No	26 (35.14%)	42 (77.78)	68 (53.13%)	<0.001
Yes	48 (64.87%)	12 (22.22)	60 (46.88%)	

The raw methylation beta values from the HumanMethylation 450k BeadChip (Illumina) are obtained *via* the Illumina Beadstudio program. Samples with probe detection call rates <90% and those with an average intensity value of either <50% of the experiment-wide sample mean or <2,000 arbitrary units (AU) are excluded from further analysis. The beta values are further converted to M-values and a total of 335,669 CpG sites are used for subsequent analysis. For the details of the data, readers are referred to the website http://gradytraumaproject.com/. The data set can be downloaded at https://www.ncbi.nlm.nih.gov/geo/query/acc.cgi?acc=GSE72680.

Lutz and Turecki (2014) reviewed human studies indicating that early-life experiences (e.g., childhood maltreatment) regulate life-long stress activities (e.g. psychopathological disorders) through epigenetic regulations (e.g., DNAms). Klengel et al. (2014) found that exposure to stress can induce long-lasting changes in DNAs, which may relate to the pathophysiology of depression and PTSD. This evidence suggests that a mediation model can help to understand how childhood maltreatment can alter long lasting DNAm changes which further affect phycological disorders such as PTSD. We fit the following mediation model while adjusting for the cell type effect whenever CpG sites are involved, i.e.,

$$\begin{array}{l} \text{logit}(P\text{)=}\theta_{1}+c_{Maktreatment}\text{Maltreatment,}\\ {\text{CpG}}_{j}={\theta^{\prime }}_{j}+a_{j}\text{Maltreatment+}\lambda_{2}^{T}+\delta_{1}^{T}\text{CellType+}\varepsilon j.j=1,\ldots ,k,\\ \text{logit(}P\text{)=}\theta_{2}+{c^{\prime }}_{Maltreatment}\text{Maltreatment}+\sum_{j=1}^{k}b_{j}CpG_{j}+\lambda_{2}^{T}\text{CellType}. \end{array}$$

**Figure 7:**
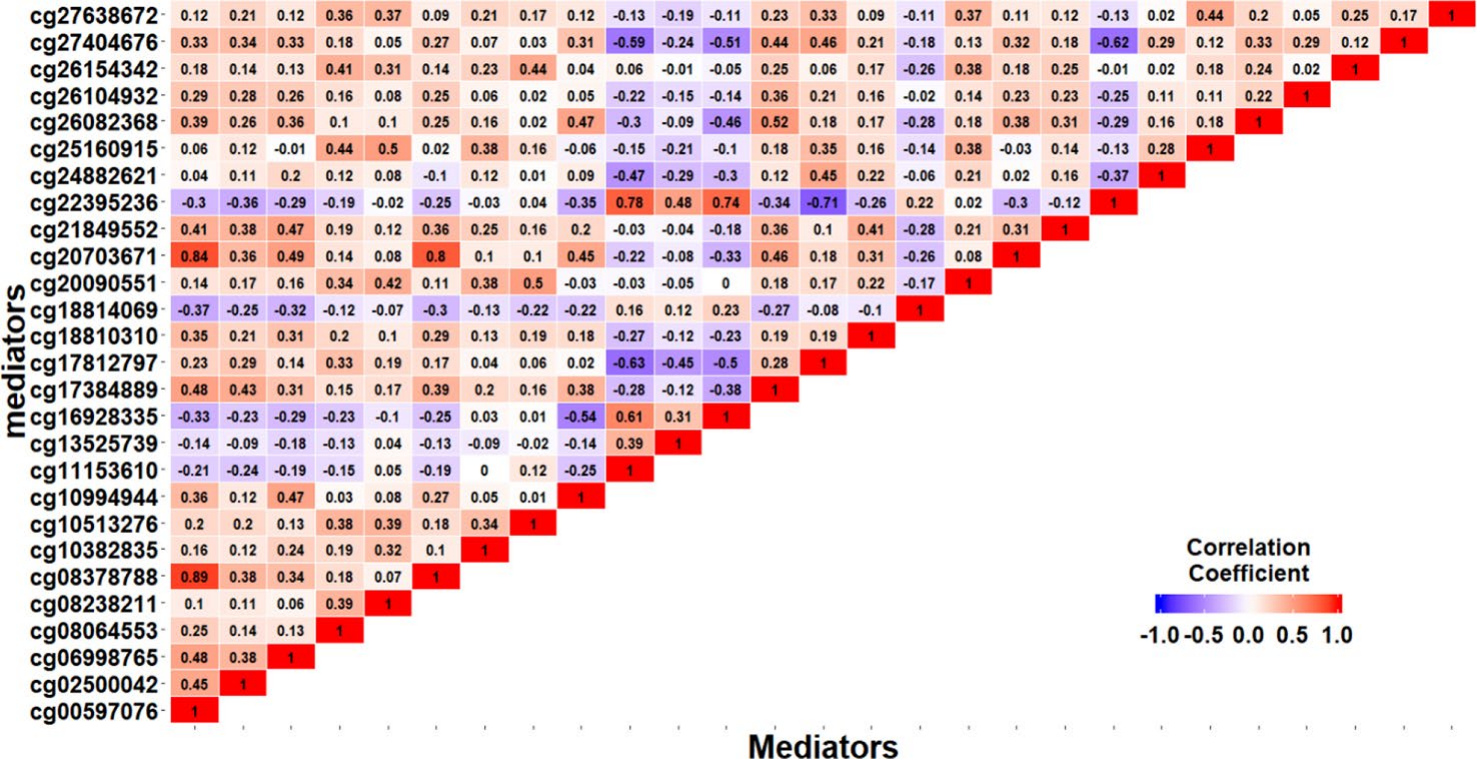
Heatmap of 27 methylation signals after screening with the SIS procedure.

Based on the first regression model, we identify an existing relationship between childhood maltreatment and PTSD with ${\hat{c}}_{Maltreament}=1.866$ (95% CI: [1.091, 2.698]) by fitting a logistic regression model. When doing the SIS step to screen CpG sites, we keep *n/*log(*n*) mediators rather than *n*/2log(*n*) to avoid missing important loci, due to the small sample size. After the SIS step, 27 DNAm sites are left in the model for further analysis. **Table 5** summarizes the results. HDMA identifies two significant CpG sites (cg06998765 and cg16928335) which reside in gene *RPS6KL1* on chromosome 12 and gene *SH2D1A* on chromosome X, respectively. The two CpG sites, cg06998765 and cg16928335, respectively explain 22.73% and 19.95% of the total mediation effect. HIMA identifies one CpG site which is a subset of what HDMA detected. A heatmap of the 27 methylation signals after SIS is shown in **Figure 7**. It is clear that there are strong correlations between some CpG sites and it is not surprising that HDMA identified one more CpG site since it can handle correlation well. We further test the methylation signal difference between cases and controls for the two CpG sites and the results show significant differences for cg06998765 (*t* = 4.109, *P*<0.001) and cg16928335 (*t* = 2.242, *P* = 0.027).

**Table 5:** List of significant CpGs identified by HDMA and HIMA.

Method	CpG	Chr	Gene name	$\hat{a}$	$\hat{b}$	$\hat{a}\hat{b}$	% of total effect	*p*-value
HDMA	cg06998765	12	RPS6KL1	0.266	1.594	0.424	22.748	0.020
	cg16928335	X	SH2D1A	−0.222	−1.674	0.372	19.933	0.046
HIMA	cg06998765	12	RPS6KL1	0.266	0.535	0.142	7.635	0.030

**Figure 8:**
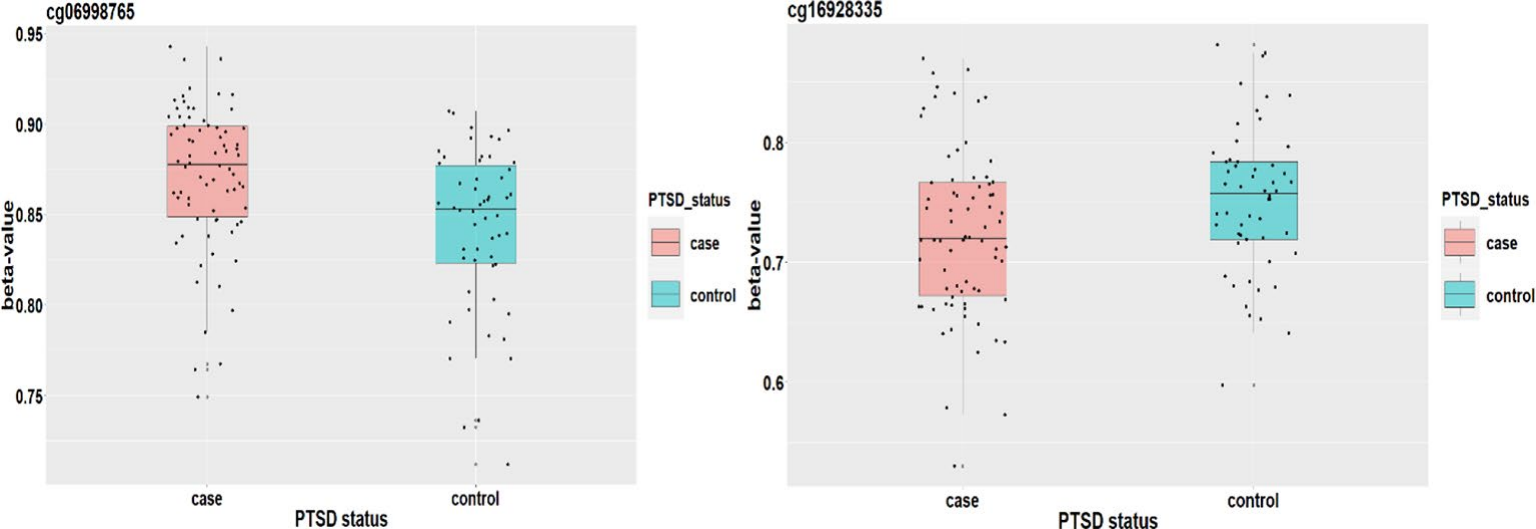
The DNAm status (*β* value) of cg06998765 and cg16928335 between PTSD cases and controls.

**Figure 8** plots the methylation signals between cases and controls for the two CpG sites. Ward et al. (2017) applied a genome-wide analysis method to analyze UK Biobank data and identified four loci associated with mood instability. Gene *RPS6KL1* is located nearby one of these regions, suggesting a potential role of this DNAm on PTSD. Although we cannot find evidence to support the association between PTSD and gene *SH2D1A* where cg06998765 is located, a two samples t-test shows that there is a significant difference on methylation signal of cg06998765 between cases and controls. The upshot suggests that this CpG site may have an important role to mediate the effect of childhood maltreatment on PTSD (**Figure 8**).]

## Discussion

A large body of literature has suggested that environmental exposures can leave epigenetic tags such as DNAm changes which further affect disease risks. Such a causal relationship can be better understood with a causal mediation model, with the hope to identify important epigenetic players (e.g., DNAm) that mediate the relationship between an exposure and a disease outcome. As biotechnology getting cheaper and cheaper, the pace of generating epigenetic data becomes faster and faster. In many applications, the number of epigenetic features can be much larger than the sample size, resulting in the so-called (ultra-) high dimensional data. These high-dimensional data provide unprecedented opportunity to reveal the molecular mechanism of many diseases. In the meantime, they also challenge the traditional mediation analysis methods which are developed for low-dimensional data.

In this work, we propose a high-dimensional mediation model to tackle issues due to high dimensionality and high correlation. Different from the HIMA approach developed by Zhang et al. (2016), our method is built under a high-dimensional inference framework where we can simultaneously estimate and test the effect of regression coefficients in a regression model. The high-dimensional testing method implements a debias approach and the de-sparsified estimates can well take care of correlations between mediators (Zhang and Zhang, 2014). Such correlations are naturally arising due to the nature of the epigenetic data. We illustrate the performance of the proposed method *via* simulations and case studies and compare with the HIMA method (Zhang et al, 2016). The simulation studies show that our method (HDMA) outperforms the HIMA method when there are high correlations between mediators. Thus, HDMA can be safely used in a high-dimensional mediation analysis from population studies.

In the first real data analysis, four CpG sites are identified to mediate the effects between alcohol consumption and EOC status. HDMA identifies two more CpG sites than HIMA does. In the second real data analysis, of the two CpG sites identified by HDMA, one overlaps with HIMA. These CpG sites may mediate the effect of childhood maltreatment to PTSD risk in adulthood. In both real data analysis, HDMA identifies more CpG sites than HIMA does, demonstrating the superior power of HDMA over HIMA. However, further biological verification is needed to validate the results, since statistical significance does not guarantee a biological significance.

Philibert et al. (2012) found that alcohol intake is linked to widespread changes in DNAm in women. Cvetkovic (2003) showed that DNAm alterations are an early step in carcinogenesis and could represent a mechanism of disease. Many such pieces of evidence point to the proper linkage of DNAm mediating the relationship between alcohol consumption and EOC status. Similar evidence also supports the linkage between childhood maltreatment and PTSD mediated by DNAm. Mehta et al. (2013) provided epigenetic support that childhood maltreatment is likely to carve long-lasting epigenetic marks, leading to adverse health outcomes such as PTSD in adulthood. Childhood abuse can increase the risk of neuropsychiatric and cardiometabolic disease via changes in epigenetic marks (Szyf, 2012; Yang et al., 2013). These studies support the mediation role of DNAm between childhood maltreatment and the risk of developing PTSD in adulthood.

The mediation effect in this study is based on a linear effect assumption, while effects such as interactions including magnitude epistasis and sign epistasis are not considered. Such kinds of complex interactive mechanisms can complicate the model, especially under a high-dimensional setup. For example, if there are antagonistic epistatic interactions among mediators, the mediation effects between exposure and the outcome can be weakened, leading to the failure to detect the mediation effects. If there are synergistic epistatic interactions among mediators, the existence of mediators can produce a synergistic effect to enhance their mediation effect. In the event of multiple exposures, models can be even more complicated. Under these situations, it is not clear on how to model and assess the mediation effect in a high-dimensional setup. These issues imply the simplicity of the current method and also raise modeling challenges for further methodological development. We will take these into consideration in our future studies. The R code that implements the method can be found in github with weblink: https://github.com/YuzhaoGao/High-dimensional-mediation-analysis-R/blob/master/HDMA.R.

## Data Availability Statement

The raw data supporting the conclusions of this manuscript will be made available by the authors, to any qualified researcher. Requests to access the datasets should be directed to Yuehua Cui cuiy@msu.edu.

## Author Contributions

YG implemented the method and drafted the manuscript. HY and RF were involved in the data analysis. YZ and EG participated in the study. YC conceived the idea, designed the study, and drafted the manuscript. All authors read and approved the final manuscript.

## Conflict of Interest

The authors declare that the research was conducted in the absence of any commercial or financial relationships that could be construed as a potential conflict of interest.

## References

[B1] Abdolmaleky H. M. Smith C. L. Faraone S. Shafa R. Stone W. Glatt S. J. (2004). Methylomics in psychiatry: modulation of gene-environment interactions may be through DNA methylation. Am. J. Med. Genet. Part B (Neuropsychiatric Genetics) 127B, 51–59. 10.1002/ajmg.b.20142 15108180

[B2] Adalsteinsson B. T. Gudnason H. Aspelund T. Harris T. B. Launer L. J. Eiriksdottir G. (2012). Heterogeneity in white blood cells has potential to confound DNA methylation measurements. PLoS One, 7 (10), 1–9. 10.1371/journal.pone.0046705 PMC3465258 23071618

[B3] Adalsteinsson B. T. Gudnason H. Aspelund T. (2012). Heterogeneity in white blood cells has potential to confound DNA methylation measurements. PLoS One 7 (10), 1–9. 10.1371/journal.pone.0046705 PMC3465258 23071618

[B4] Auner V. Kriegshäuser G. Tong D. Horvat R. Reinthaller A. Mustea A. (2009). KRAS mutation analysis in ovarian samples using a high sensitivity biochip assay. BMC Cancer 9, 1–8. 10.1186/1471-2407-9-111 19358724 PMC2671522

[B5] Baron R. M. Kenny D. A. (1986). The moderator-mediator variable distinction in social psychological research: conceptual, strategic, and statistical Considerations. J. Pers. Soc. Psychol 51 (6), 1173–1182 3806354 10.1037//0022-3514.51.6.1173

[B6] Beck A. T. Ward C. H. Mendelson M. Mock J. Erbaugh J. (1961). Inventory for measuring depression. Arch. Gen. Psychiatry 4 (6), 561–571. 10.1001/archpsyc.1961.01710120031004 13688369

[B7] Berger R. L. Hsu J. C. (1996). Bioequivalence trials, intersection-union tests and equivalence confidence sets. Stat. Sci. 11 (4), 283–319. 10.1214/ss/1032280304

[B8] Cvetkovic D. (2003). Early events in ovarian oncogenesis. Reprod. Biol. Endocrin. 1, 68. 10.1186/1477-7827-1-68 PMC239895 14577833

[B9] Dezeure R. Bühlmann P. Meier L. Meinshausen N. (2015). High-dimensional inference: confidence intervals, p-values and R-software hdi. Stat. Sci. 30 (4), 533–558. 10.1214/15-STS527

[B10] Dongen J. Nivard M. G. Willemsen G. Hottenga J. Helmer Q. Dolan C. V. (2016). Genetic and environmental influences interact with age and sex in shaping the human methylome. Nat. Commun. 7, 1–13. 10.1038/ncomms11115 PMC4820961 27051996

[B11] E. Shrout P. Bolger N. (2002). Mediation in experimental and nonexperimental studies: new procedures and recommendations. Psychol. Meth. 7 (4), 422–445. 10.1037//1082-989X.7.4.422 12530702

[B12] Elgaaen B. V. Haug K. B. F. Wang J. Olstad O. K. Fortunati D. Onsrud M. (2010). POLD2 and KSP37 (FGFBP2) correlate strongly with histology, stage and outcome in ovarian carcinomas. PLoS One, 5 (11), e13837. 10.1371/journal.pone.0013837 21079801 PMC2973954

[B13] Fan J. Lv J. (2008). Sure independence screening for ultrahigh dimensional feature space. J. R. Stat. Soc. Ser. B: Stat. Methodol. 70 (5), 849–911. 10.1111/j.1467-9868.2008.00674.x PMC2709408 19603084

[B14] Foa E. B. Tolin D. F. (2000). Comparison of the PTSD symptom scale-interview version and the clinician-administered PTSD scale. J. Trauma. Stress 13 (2), 181–191. 10838669 10.1023/A:1007781909213

[B15] Guida F. Sandanger T. M. Castagné R. Campanella G. Polidoro S. Palli D. (2015). Dynamics of smoking-induced genome-wide methylation changes with time since smoking cessation. Hum. Mol. Genet. 24 (8), 2349–2359. 10.1093/hmg/ddu751 25556184 PMC4380075

[B16] Hafeman D. M. Schwartz S. (2009). Opening the black box: a motivation for the assessment of mediation. Int. J. Epidemiol. (38), 838–845. 10.1093/ije/dyn372 19261660

[B17] Huang Y.-T. VanderWeele T. Lin X. (2014). Joint analysis of SNP and gene expression data in genetic association studies of comples disease. Ann. Appl. Stat. 8 (1), 352–376. 10.1214/13-AOAS690 24729824 PMC3981558

[B18] Huang Y. Liang L. Moffatt M. F. Cookson W. O. C. Lin X. (2015). iGWAS:integrative genome-wide association studies of genetic and genomic data for disease susceptibility using mediation analysis. Genet. Epidemiol. 39 (5), 347–356. 10.1002/gepi.21905 25997986 PMC4544880

[B19] Huang Y. Pan W. (2016). Hypothesis test of mediation effect in causal mediation model with high-dimensional continuous mediators. Biometrics, (72), 402–413. 10.1111/biom.12421 26414245

[B20] Houseman E. A. Accomando W. P. Koestler D. C. Christensen B. C. Marsit C. J. Nelson H. H. (2012). DNA methylation arrays as surrogate measures of cell mixture distribution. BMC Bioinformatics 13, 86. 10.1186/1471-2105-13-86 22568884 PMC3532182

[B21] Imai K. Keele L. Tingley D. (2010). A general approach to causal mediation analysis. Psychol. Meth. 15 (4), 309–334. 10.1037/a0020761 20954780

[B22] Klengel T. Pape J. Binder E. B. Mehta D. (2014). The role of DNA methylation in stress-related psychiatric disorders. Neuropharmacology 80, 115–132. 10.1016/j.neuropharm.2014.01.013 24452011

[B23] Klengel T. Mehta D. Anacker C. Rex-haffner M. Pruessner J. C. Pariante C. M. (2016). Allele-specific FKBP5 DNA demethylation mediates gene-childhood trauma interactions. Cornell Law Rev. 101 (6), 1533–1595. 10.1038/nn.3275 PMC4136922 23201972

[B24] Koestler D. C. Chalise P. Cicek M. S. Cunningham J. M. Armasu S. Larson M. C. (2014). Integrative genomic analysis identifies epigenetic marks that mediate genetic risk for epithelial ovarian cancer. BMC Medical Genomics 7 (8), 1–14. 10.1186/1755-8794-7-8 24479488 PMC3916313

[B25] Li S. Hursting S. Davis B. McLachlan J. A. Barrett J. car. (2003). Environmental exposure, DNA methylation, and gene regulation: lessons from diethylstilbesterol-induced cancers. N. Y. Acad. Sci. 983 (1), 161–169. 10.1111/j.1749-6632.2003.tb05971.x 12724221

[B26] Liu Y. Aryee M. J. Padyukov L. Fallin M. D. Hesselberg E. Runarsson A. (2013). Epigenome-wide association data implicate DNA methylation as an intermediary of genetic risk in rheumatoid arthritis. Nat. Biotechnol. 31 (2), 142–147. 10.1038/nbt.2487 23334450 PMC3598632

[B27] Lutz P. E. Turecki G. (2014). DNA methylation and childhood maltreatment: From animal models to human studies. Neuroscience 264, 142–156. 10.1016/j.neuroscience.2013.07.069 23933308 PMC5293537

[B28] MacKinnon D. P. (2008). Introduction to statistical mediation analysis. New York, London:Taylor & Francis Group.

[B29] Mehta D. Klengel T. Conneely K. N. Smith A. K. Altmann A. Pace T. W. (2013). Childhood maltreatment is associated with distinct genomic and epigenetic profiles in posttraumatic stress disorder. Proc. Nat. Acad. Sci. 110 (20), 8302–8307. 10.1073/pnas.1217750110 23630272 PMC3657772

[B30] Nakayama N. Nakayama K. Yeasmin S. Ishibashi M. Katagiri A. Iida K. (2008). KRAS or BRAF mutation status is a useful predictor of sensitivity to MEK inhibition in ovarian cancer. Br. J. Cancer 99 (12), 2020–2028. 10.1038/sj.bjc.6604783 19018267 PMC2607229

[B31] Pearl J. (2012). The causal mediation formula – a guide to the assessment of pathways and mechanisms. Soc. Prev. Res, (13), 426–436. 10.1007/s11121-011-0270-1 22419385

[B32] Pfeffer J. Devoe S. E. (2009). Economic evaluation: the effect of money and economics on attitudes about volunteering. J. Econ. Psychol. (30), 500–508. 10.1016/j.joep.2008.08.006

[B33] Philibert R. A. Plume J. M. Gibbons F. X. Brody G. H. Beach S. R. H. (2012). The impact of recent alcohol use on genome wide DNA methylation signatures. Front. Genet. 3 (APR), 1–8. 10.3389/fgene.2012.00054 22514556 PMC3322340

[B34] Pierce B. L. Tong L. Chen L. S. Rahaman R. Argos M. Jasmine F. (2014). Mediation analysis demonstrates that trans-eQTLs are often explained by cis-mediation: a genome-wide analysis among 1800 South Asians. PLoS Genetics 10 (12), e1004818. 10.1371/journal.pgen.1004818 25474530 PMC4256471

[B35] Preacher K. Hayes A. (2008). Asymptotic and resampling strategies for assessing and comparing indirect effects in multiple mediator models. Behav. Res, Meth. 40 (3), 879–891. 10.3758/BRM.40.3.879 18697684

[B36] Robertson K. D. (2005). DNA methylation and human disease. Nat. Rev. Genet. 6 (8), 597–610. 10.1038/nrg1655 16136652

[B37] Rocca C. H. Doherty I. Padian N. S. Hubbard A. E. Minnis A. M. (2010). Pregnancy intentions and teenage pregnancy among latinas: A Mediation Analysis. Perspect. Sex. Reprod. Health 42 (3), 186–196. 10.1363/4218610 20887287 PMC2951312

[B38] Sobel M. E. (1982). Asymptotic confidence intervals for indirect effects in structural equation models. Sociol. Methodol. 13, 290–312. 10.2307/270723

[B39] Sun T. Zhang C. (2012). Scaled sparse linear regression. Biometrika 99 (4), 879–898. 10.1093/biomet/ass043

[B40] Szyf M. (2012). The early-life social environment and DNA methylation. Clin. Genet. (81), 341–349. 10.1111/j.1399-0004.2012.01843.x 22236068

[B41] Tibshiranit R. (1996). Regression shrinkage and selection *via* the lasso. J. R. Stat. Soc. Ser. B: Stat. Methodol. (1), 267–288. 10.1111/j.2517-6161.1996.tb02080.x

[B42] Titus A. J. Gallimore R. M. Salas L. A. Christensen B. C. (2017). Cell-type deconvolution from DNA methylation: a review of recent applications. Hum. Mol. Genet. 26 (R2), R216–R224. 10.1093/hmg/ddx275 28977446 PMC5886462

[B43] Uddin M. Galea S. Chang S. C. Aiello A. E. Wildman D. E. De Los Santos R. (2011). Gene expression and methylation signatures of MAN2C1 are associated with PTSD. Dis. Markers 30 (2–3), 111–121. 10.3233/DMA-2011-0750 21508515 PMC3188659

[B44] Van De Geer S. Bühlmann P. Ritov Y. Dezeure R. (2014). On asymptotically optimal confidence regions and tests for high-dimensional models. Ann. Stat. 42 (3), 1166–1202. 10.1214/14-AOS1221

[B45] Vanderweele T. J. Vansteelandt S. (2010). Odds ratios for mediation analysis for a dichotomous outcome. Am. J. Epidemiol. 172 (12), 1339–1348. 10.1093/aje/kwq332 21036955 PMC2998205

[B46] Varela-Rey M. Woodhoo A. Martinez-Chantar M.-L. Mato J. Lu S. C. (2013). Alcohol, DNA methylation, and cancer. Alcohol Research 35, 25–35. 10.4067/S0370-41062008000700008 24313162 PMC3860423

[B47] Wang T. Samworth R. J. (2018). High dimensional change point estimation via sparse projection. J. R. Stat. Soc. Ser. B: Stat. Methodol. 80 (1), 57–83. 10.1111/rssb.12243

[B48] Ward J. Strawbridge R. J. Bailey M. E. S. Graham N. Ferguson A. Lyall D. M. (2017). Genome-wide analysis in UK Biobank identifies four loci associated with mood instability and genetic correlation with major depressive disorder, anxiety disorder and schizophrenia. Trans. Psychiatry 7, 1264. 10.1038/s41398-017-0012-7 PMC5802589 29187730

[B49] Wingo A. P. Velasco E. R. Florido A. Lori A. Choi D. C. Jovanovic T. (2018). Expression of the PPM1F gene is regulated by stress and associated with anxiety and depression. Biol. Psychiatry 83 (3), 284–295. 10.1016/j.biopsych.2017.08.013 29054677 PMC5743606

[B50] Wu D. Yang H. Winham S.J. Natanzon Y. Koestler D.C. Luo T. (2018). Mediation analysis of alcohol consumption, DNA methylation, and epithelial ovarian cancer. J. Hum. Genet. 63, 339–348. 10.1038/s10038-017-0385-8 29321518 PMC5985822

[B51] Yang B. Zhang H. Ge W. Weder N. Douglas-palumberi H. Perepletchikova F. (2013). Child abuse and epigenetic mechanisms of disease risk. Am. J. Prev. Med. 44 (2), 101–107. 10.1016/j.amepre.2012.10.012 23332324 PMC3758252

[B52] Zhang X. Cheng G. (2017). Simultaneous Inference for High-Dimensional Linear Models. J. Am. Stat. Assoc. 112 (518), 757–768. 10.1080/01621459.2016.1166114

[B53] Zhang Z. Wang L. (2013). Methods for mediation analysis with missing data. Psychom. Soc. 78 (1), 154–184. 10.1007/s11336-012-9301-5 25107523

[B54] Zhang C. Zhang S. S. (2014). Confidence intervals for low dimensional parameters in high dimensional linear models. J. R. Stat. Soc. Ser. B: Stat. Methodol. 76 (1), 217–242. 10.1111/rssb.12026

[B55] Zhang H. Zheng Y. Zhang Z. Gao T. Joyce B. Yoon G. (2016). Estimating and testing high-dimensional mediation effects in epigenetic studies. Bioinformatics 32 (20), 3150–3154. 10.1093/bioinformatics/btw351 27357171 PMC5048064

[B56] Zou H. Hastie T. (2005). Regularization and variable selection via the elastic net. J. R. Stat. Soc. Ser. B: Stat. Methodol. 67 (2), 301–320. 10.1111/j.1467-9868.2005.00503.x

[B57] Zou H. (2006). The adaptive lasso and its oracle properties. J. Am. Stat. Assoc. 101 (476), 1418–1429. 10.1198/016214506000000735

